# Self-assembled hyaluronan nanocapsules for the intracellular delivery of anticancer drugs

**DOI:** 10.1038/s41598-019-47995-8

**Published:** 2019-08-09

**Authors:** Ana Cadete, Ana Olivera, Magnus Besev, Pradeep K. Dhal, Lídia Gonçalves, António J. Almeida, Guillaume Bastiat, Jean-Pierre Benoit, María de la Fuente, Marcos Garcia-Fuentes, María José Alonso, Dolores Torres

**Affiliations:** 1 Nanobiofar Group, IDIS, CIMUS, University of Santiago de Compostela, Santiago de Compostela, Spain; 2 Department of Pharmaceutics and Pharmaceutical Technology, School of Pharmacy, University of Santiago de Compostela, Santiago de Compostela, Spain; 3 Sanofi-Global R&D, Sanofi US, Waltham, USA; 4 Research Institute for Medicines (iMed.ULisboa), Faculty of Pharmacy, Universidade de Lisboa, Lisbon, Portugal; 5 Micro et Nanomedecines Translationnelles, MINT, Université Angers, INSERM 1066, CNRS 6021, 4 rue Larrey, Angers, France; 6 Nano-Oncology Unit, Translational Medical Oncology Group, Health Research Institute of Santiago de Compostela (IDIS), SERGAS, Santiago de Compostela, Spain; 7 Cancer Network Research (CIBERONC), Madrid, Spain

**Keywords:** Cell delivery, Chemical modification, Drug delivery, Nanoparticles

## Abstract

Preparation of sophisticated delivery systems for nanomedicine applications generally involve multi-step procedures using organic solvents. In this study, we have developed a simple self-assembling process to prepare docetaxel-loaded hyaluronic acid (HA) nanocapsules by using a self-emulsification process without the need of organic solvents, heat or high shear forces. These nanocapsules, which comprise an oily core and a shell consisting of an assembly of surfactants and hydrophobically modified HA, have a mean size of 130 nm, a zeta potential of −20 mV, and exhibit high docetaxel encapsulation efficiency. The nanocapsules exhibited an adequate stability in plasma. Furthermore, *in vitro* studies performed using A549 lung cancer cells, showed effective intracellular delivery of docetaxel. On the other hand, blank nanocapsules showed very low cytotoxicity. Overall, these results highlight the potential of self-emulsifying HA nanocapsules for intracellular drug delivery.

## Introduction

Nanomedicine research aims at developing therapeutic agents to address chronic and serious human diseases. Progress in nanomedicine research for cancer therapy has provided new opportunities for the development of multi-functional nanocarriers, with the potential to improve tissue and organ specific intracellular delivery of anticancer drugs with minimal off target toxicity^1,2^. Versatility of nanomaterials enables one to design nanocarriers of diverse physical and chemical properties to achieve specific structures and functions. Nanocapsules, which comprise an oily core surrounded by a polymer shell, have gained special attention due to their versatile structures and tunable physicochemical properties^3,4^. The oil core has the ability to efficiently encapsulate hydrophobic molecules, while the polymeric shell endows the carrier with desirable pharmaceutical characteristics, such as drug protection, extended stability and targeting^5^. The rational choice of starting materials is key to create nanocapsules with optimal physicochemical characteristics, low toxicity, and high loading capacity to target and kill cancer cells^6^.

Hyaluronic acid (HA) is a unique and versatile anionic natural polymer, which is ubiquitous in nature. It is produced by virtually every tissue in higher organisms and some bacteria. High molecular weight HA that is free of contaminating proteins and nucleotides is non-immunogenic. Presence of a large number of carboxyl and hydroxyl groups in HA allows facile chemical modification to conjugate bioactive agents^7,8^. Furthermore, HA is a known ligand of CD44 receptors that are overexpressed in many tumor types. These features combined with its biodegradability make HA an attractive building block to prepare drug loaded nanocapsules^9^.

Previously, we have developed HA-based nanocapsules by using a solvent displacement method where the negatively charged HA shell was associated with a cationic surfactant by electrostatic interactions^10^. Chemically modified HA carrying hydrophobic side chains could be an interesting alternative to prepare nanocapsules. Such a polymer is expected to self-assemble without the need for any cationic surfactants, which could lead to elimination of inherent toxicity of cationic surfactants^11^. With respect to imparting desired hydrophobicity, HA derivatives can be prepared with appropriate degree of substitution, without affecting its receptor targeting characteristics^12^. These amphiphilic HA derivatives have already been used for the formation of micelles with the help of sonication^13–15^ or electrostatic interactions^16^ for the encapsulation of hydrophobic drugs like doxorubicin. However, this is the first report on the use of hydrophobically modified HA to prepare nanocapsules by a self-emulsifying procedure. Since these polymeric nanocapsules can be prepared without using organic solvents, heat or mechanical stirring, this offers an attractive method to incorporate thermosensitive molecules including peptides, proteins, and antibodies^17^.

Self or spontaneous emulsification is a low energy method mostly described for the preparation of nanoemulsions^18–22^. Using this process, the formation of nanosized droplets is mainly dependent on the modulation of the interfacial phenomenon and the intrinsic physicochemical properties of oils and surfactants^23^. In the same way as nanoemulsions, polymeric nanocapsules can be prepared without organic solvents, heat or mechanical stirring, providing advantages from the manufacturing and scale-up standpoint. Moreover, the development of formulation techniques with less organic solvents and lower energy levels is expected to have a positive impact in the environment, as well as on the final production costs^24^. In this manuscript, we report for the first time the use of dodecyl side chain containing HA as the precursor polymer to prepare nanocapsules by a spontaneous emulsification process. Docetaxel was successfully loaded in HA-based nanocapsules. Potential therapeutic utility of these nanocapsules to further improve intracellular delivery of docetaxel was evaluated *in vitro* by using A549 lung cancer cells.

## Results

### Synthesis and characterization of dodecylamide-functionalized sodium hyaluronate

**Figure 1:**
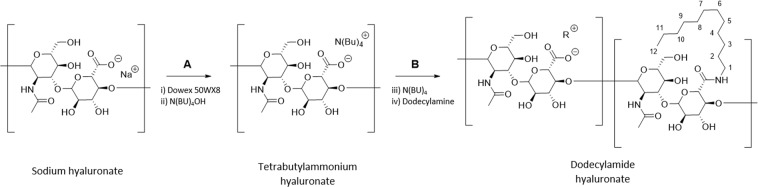
Reaction scheme for the synthesis of dodecylamide functionalized sodium hyaluronate. (**A**) (i) Dowex 50WX8-400 (ii) tetrabutylammonium hydroxide. (**B**) (i) 2-bromo-1-ethyl pyridinium tetrafluoroborate (BEP), dodecylamine (ii) Dowex 50WX8-400 (iii) Sodium Hydroxide.

Dodecylamide-functionalized sodium hyaluronate (C12-HA) was synthesized by reacting tetrabutylammonium salt of HA with 1-aminododecane by using 2-bromo-1-ethyl pyridinium tetrafluoroborate as the amide coupling reagent. The reaction process is shown in Fig. 1.

**Figure 2:**
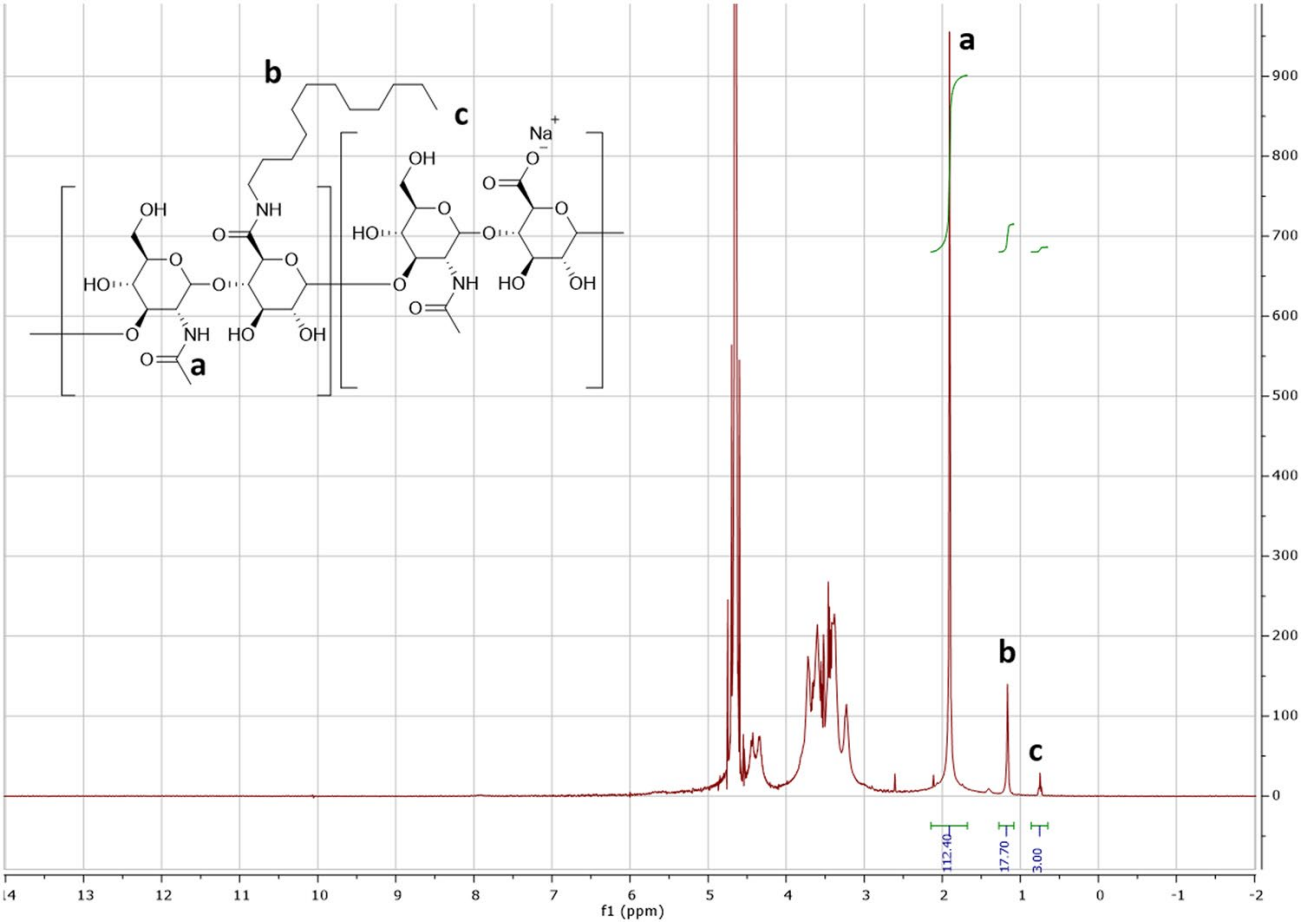
Representative ^1^H NMR (400 MHz) spectrum of dodecylamide functionalized sodium hyaluronate in D_2_O.

The reaction yield for each synthetic step was between 50–70%. C12-HA was characterized by ^1^H NMR spectroscopy using a Bruker Avance 400 MHz NMR spectrometer. A representative NMR spectrum, taken at 30 °C, is shown in Fig. 2. Analysis of the spectrum confirms the structure and composition of the polymer. For example, the peak at 1.93 ppm (a) corresponds to the methyl protons of the acetamido moiety of HA. Furthermore, the peaks at 1.22 ppm and 0.92 ppm correspond to the methylene ((CH_2_)_10_) and terminal methyl protons, respectively of the dodecylamide side chain. The degree of substitution (DS) of the dodecylamide group was determined from the peak area ratio of the methyl groups of the acetamide group of HA (a) and the methyl group of dodecylamide substituent (c) (Fig. 2). The DS of e different lots was found to be in the range of 2.5% to 5.0%. The synthesis process has been repeated several times and has been found to be highly reproducible.

### Preparation of HA nanocapsules

In the first step, the experimental conditions required for the formation of a nanoemulsion (without the HA shell) without using organic solvents, high temperature or shear forces were investigated. The components for preparing such nanoemulsions included Miglyol®812 and Tween®80 for the oil phase and Solutol®HS15 in the aqueous solution. Formulation optimization was carried out by varying the following components: (1) the concentration of Solutol®HS15 solution, (2) the ratio between Miglyol®812 and Tween®80 in the oil phase, and (3) the ratio between the oil and the aqueous phases. The results (shown in Table 1) show that the size of the nanoemulsions ranged between 138–169 nm with a polydispersity index of ≤0.2.

**Table 1:** Effect of variation in experimental parameters on the physicochemical properties of the nanoemulsions prepared by self-emulsification.

Formulation variables			NEs characterization*
Solutol®HS15 conc. (mg/mL)	Miglyol®812/Tween®80 ratio (w/w)	Oil/aq. phase ratio (v/v)	Size (nm)
2.5	1:1	1:2	138 ± 3
5			138 ± 2
15			149 ± 3
25			140 ± 1
**2**.**5**	**1:1**	1:2	138 ± 3
	**1**.**5 :1**		147 ± 3
	**2 :1**		164 ± 1
	**3**.**5 :1**		159 ± 3
2.5	1:1	**1:3**	139 ± 2
		**1:4**	144 ± 1
		**1:5**	152 ± 3
		**1:8**	138 ± 3

*Polydispersity index was 0.2 for all formulations except 3.5:1 Miglyol®812/Tween**®**80 ratio which was 0.3.

The concentration of Solutol®HS15 in the aqueous phase did not significantly change the globule size and the polydispersity index. Therefore, the smallest concentration (2.5 mg/mL) was fixed for subsequent studies intended to analyze the effect of Miglyol®812/Tween®80 (w/w) and the oil/aqueous phase ratios (v/v). The Miglyol®812/Tween®80 (w/w) ratio selected was 1:1 since it led to the smallest particle size (Table 1). Subsequently, the oil/aqueous phase ratio was varied from 1:1 to 1:8 (v/v) without affecting the physicochemical properties of the nanoemulsions.

Based on the above findings, the following conditions were used for the formulation of nanoemulsions: the oil phase was composed of Miglyol®812/ Tween®80 in a ratio 1:1 (w/w) and the aqueous phase of a Solutol®HS15 solution of 2.5 mg/mL. The oil phase was poured into the aqueous phase in a ratio 1:8 (v/v) and stirred at 900 rpm during 20 min.

For the nanocapsules formation, two different approaches were employed depending on the outer layer material. For unmodified HA NCs, the polymer was attached by electrostatic forces, so the oily core was previously cationized by adding the cationic surfactant Cetyl trimethylammonium bromide (CTAB) to the oily phase. In the case of nanocapsules with a C12-HA layer, we took advantage of the amphiphilic nature of this material, which enabled us to avoid the use of any cationic surfactant.

**Figure 3:**
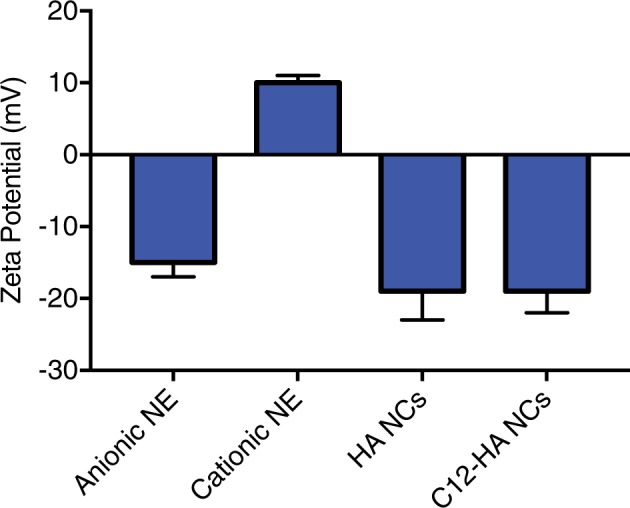
Surface charge of different nanocarriers prepared by spontaneous emulsification. Shift in the zeta potential of anionic to cationic nanoemulsions (NE) was achieved by CTAB addition (0.15 mg/mL). The addition of HA (0.25 mg/mL) or C12-HA (0.5 mg/mL) to the aqueous phase led to inversion in the charge to negative values.

Cationic nanoemulsions, used as cores for HA NCs, were initially prepared by adding different amounts (0.05, 0.10 and 0.15 mg/mL) of CTAB to the oil phase. The cationic surfactant promoted an inversion of the negatively charged nanoemulsion to positively charged one. Furthermore, increasing the amount of CTAB resulted in a higher zeta potential, without influencing the mean droplet size (See Supplementary Table S1). Since no further zeta potential increase was observed with additional CTAB in the formulation, the concentration of this surfactant was fixed at 0.15 mg/mL to prepare NCs based on unmodified HA. The formation of the HA shell around the oily nano-cores by adding 0.25 mg/ml of HA to the water phase resulted in a shift of the zeta potential from +10 mV to −19 mV (Fig. 3). When the C12-HA derivative was used to form the nanocapsules, the physicochemical properties of the system did not change appreciably (Table 2). However, higher concentrations of C12-HA were required to change the zeta potential as compared to those required previously with unmodified HA. As such, a concentration of 0.5 mg/mL of C12-HA was needed to give a negative charge around −20 mV to the nanocapsules (Fig. 3). Details on the composition and characterization of both types of nanocapsules are shown in Table 2.

**Table 2:** Composition and physicochemical characterization of HA NCs and C12-HA NCs prepared by self-emulsification.

Composition & characterization	HA NCs	C12-HA NCs
	(mg/mL)	(mg/mL)
Miglyol^®^812	59	59
Tween^®^80	58	58
Solutol^®^HS15	2.5	2.5
CTAB	0.15	—
HA	0.25	—
C12-HA	—	0.5
Size (nm)	137 ± 11	126 ± 5
PDI	0.2	0.2
Zeta Potential (mV)	−19 ± 1	−20 ± 2

**Figure 4:**
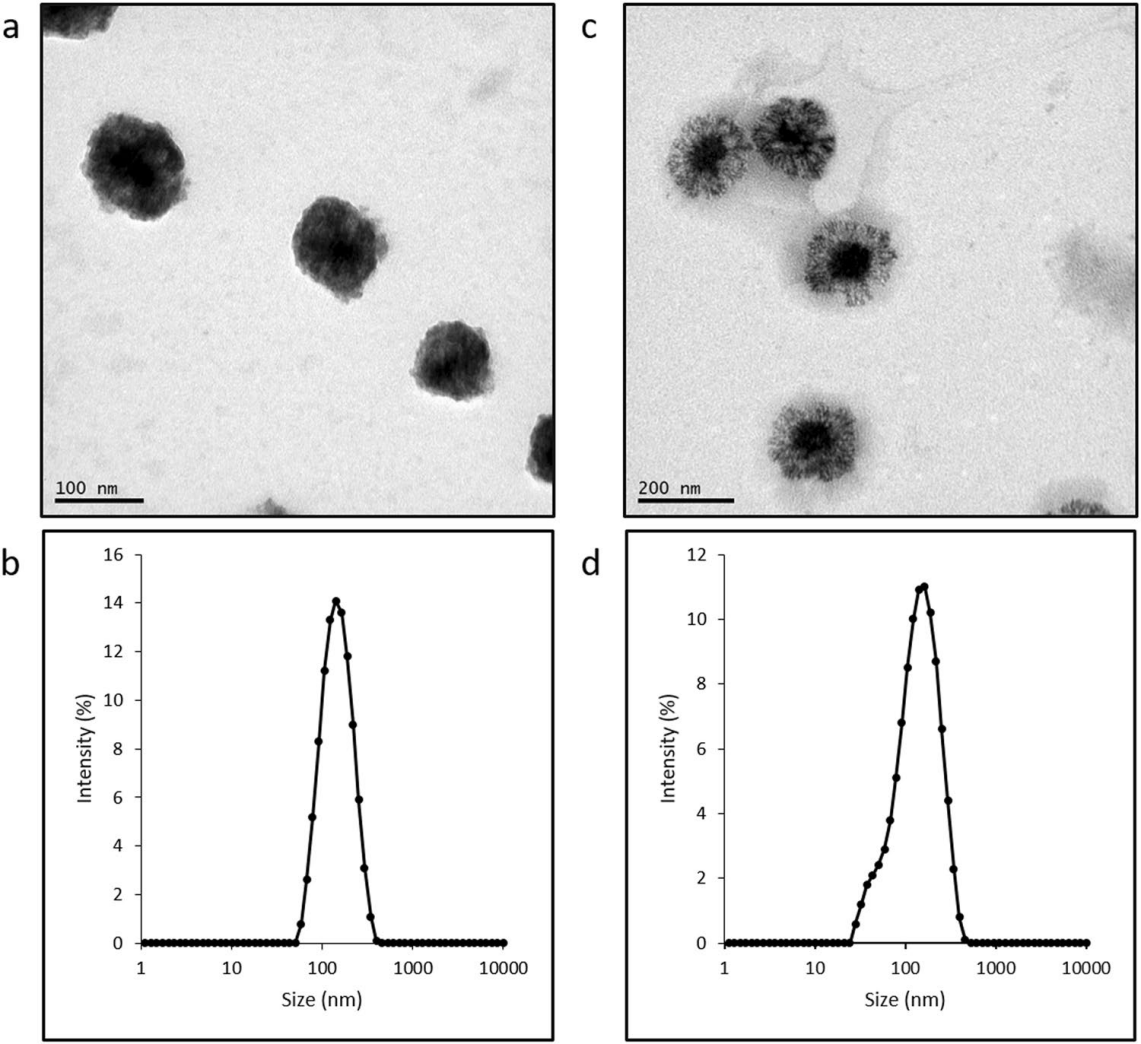
TEM images of HA nanocapsules (**a**,**c**), and the corresponding size distribution by DLS (**b**,**d**). (**a**,**b**) HA NCs; (**c**,**d**) C12-HA NCs.

Size distribution and TEM images (Fig. 4) confirmed the proposed morphology of HA NCs consisting of a monodisperse system composed of an oil core surrounded by a polymeric shell.

The stability of both HA-based nanocapsules was tested under storage conditions at 4 °C for 6 months. Under these conditions, both formulations were found to be very stable, without significant changes in particle size, polydispersity index, or zeta potential for up to 6 months (See Supplementary Table S2).

### Stability of HA nanocapsules in human plasma

**Figure 5:**
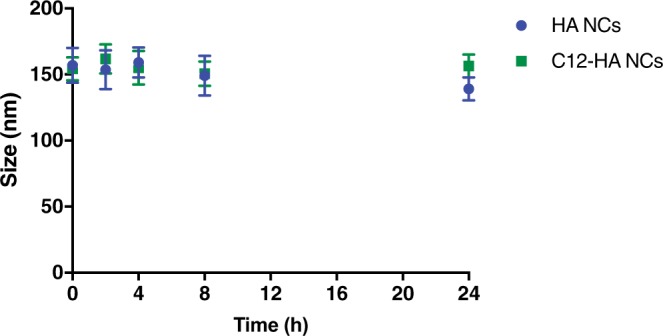
Evolution of the mean particle size of HA and C12-HA NCs incubated with human plasma, at 37 °C for 24 h.

Unmodified HA and C12-HA based NCs were incubated in human plasma at 37 °C. Both types of nanocapsules maintained their sizes upon incubation for up to 24 h (Fig. 5).

### Preparation and characterization of docetaxel-loaded HA nanocapsules

The solubility of docetaxel (DCX) in Miglyol®812 was 2.0 ± 0.2 mg/mL at room temperature. As shown in Table 2, the encapsulation efficiency was close to 90% and the production yield ~90% (Table 3). Importantly, such high encapsulation values did not affect the size and PDI of the nanocapsules.

**Table 3:** Characterization of DCX-loaded HA NCs and C12-HA NCs after purification by SEC.

Formulation	Size (nm)	PDI	ZP (mV)	EE%	Yield %
HA NCs	140 ± 5	0.2	−18 ± 2	88 ± 9	93 ± 2
C12-HA NCs	145 ± 6	0.2	−20 ± 1	86 ± 3	88 ± 8

### *In vitro* release assays

**Figure 6:**
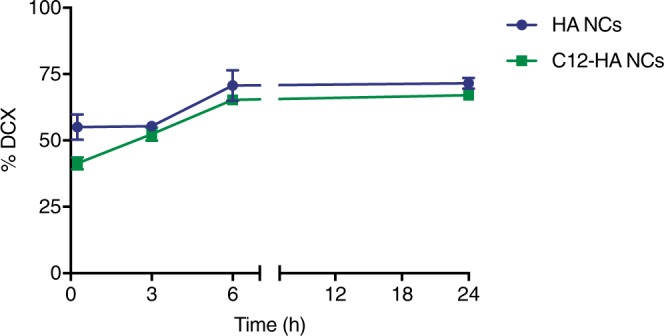
Release profile of DCX from DCX-loaded HA NCs and DCX-loaded C12-HA NCs in PBS at 37 °C for 24 h.

The release profile of DCX from the nanocapsules was assessed by a method based on the drug transfer to an oily compartment^25^. DCX was released from HA NCs and C12 HA NCs following a biphasic profile, showing an initial burst release of 55% and 45%, respectively, followed by a sustained release for 24 h (Fig. 6).

### *In vitro* cytotoxicity of empty and DCX-loaded HA nanocapsules

**Figure 7:**
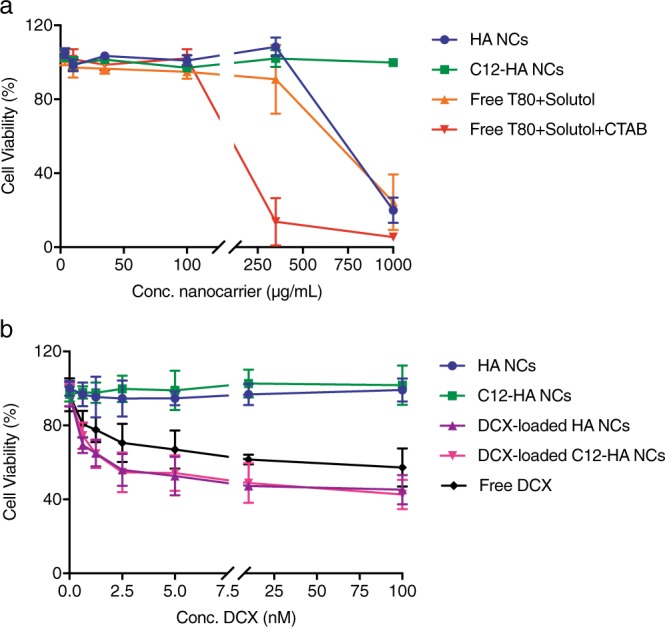
*In vitro* cell toxicity of A549 cells after (**a**) exposition to different concentrations of empty HA NCs, C12-HA NCs and free surfactants mixture for 72 h, and (**b**) free DCX, DCX-loaded HA nanocapsules and empty HA nanocapsules for 48 h.

Cytotoxicity of empty and loaded HA and C12-HA NCs was assessed against A549 lung adenocarcinoma cells after 72 h and 48 h of incubation, respectively. A long incubation time (up to 72 h) was chosen to ensure that cells would have sufficient time to multiply and to correctly measure cell activities attributed to cellular maintenance and survival^26^. Moreover, the totoxicity of the two surfactant solutions used to prepare the nanocapsules was also assessed. Both types of nanocapsules preserved the cell viability when tested at concentrations up to 350 μg/mL (Fig. 7a). Furthermore, C12-HA NCs showed no sign of cellular cytotoxicity even when tested at the highest concentration (1,000 µg/mL). On the contrary, a significant cytotoxicity was observed for the free surfactant mixture with CTAB, where only 20% of cells survived at 350 μg/mL after 72 h.

DCX-loaded HA-based NCs showed a dose dependent cytotoxicity against A549 cells (Fig. 7b). There were no statistically significant differences between DCX-loaded nanocapsules formulations and free DCX. The minimal half inhibitory concentration (IC50) for both types of DCX-loaded nanocapsules was 10 μM DCX at 48 h. Blank nanocapsules added at the same concentration as DCX-loaded NCs showed negligible toxicity.

### Intracellular uptake of HA-based nanocapsules

**Figure 8:**
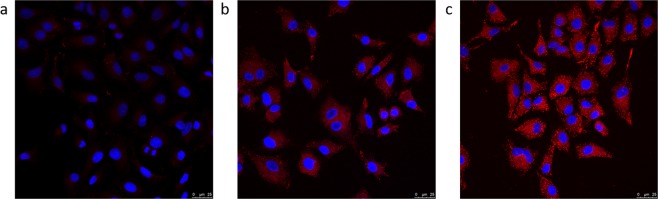
Intracellular uptake of Nile red-loaded HA-based nanocapsules in A549 cells. (**a**) Free Nile red. (**b**) Nile red-loaded HA NCs. (**c**) Nile red-loaded C12-HA NCs. Nuclei were stained with DAPI (blue). Nile Red exhibits red fluorescence.

To evaluate the intracellular uptake of HA NCs and C12-HA NCs, Nile red was loaded into both nanocapsules formulations and their cellular uptake by CD44 overexpressing A549 cells was observed by confocal microscopy. As a control, cells were exposed to a solution of the free fluorophore (same concentration as of nanocapsules), which was not internalized by the cells (Fig. 8a). On the other hand, when cells were exposed to either Nile red-loaded HA or C12-HA nanocapsules, a high fluorescence (red color) was observed at the cytoplasmic level. (Fig. 8b,c).

## Discussion

We have previously reported the ability of unmodified HA-based nanocapsules for the intracellular delivery of DCX in cancer cells^10^. These nanocapsules were formed by the solvent-displacement technique and required the use of a cationic surfactant to facilitate the attachment of the HA shell to the nanoemulsion core. The objective of this work was to adapt for the first time a spontaneous emulsification technique to the preparation of HA nanocapsules by using amphiphilic HA. Using this technology, we have successfully prepared nanocapsules of smaller size and monodisperse particle size. These nanocapsules exhibited stability in human plasma and have shown high propensity for intracellular uptake. Furthermore, the use of an amphiphilic HA derivative (dedecylamide-functionalized sodium hyaluronate, C12-HA) enabled us to prepare nanocapsules without the need of a cationic surfactant, thereby improving the toxicity profile of the resulting delivery system.

By keeping the degree of substitution in C12-HA in the range of 2.5–5%, the amphiphilic HA was soluble in water. Furthermore, such polymers retain the rheological and biodegradability properties of native HA^27^. Self-emulsifying HA nanocapsules were prepared by spontaneous emulsification after careful screening of formulation conditions. Due to the absence of organic solvents, heat or high mechanical shearing, the spontaneous emulsification process is mainly determined by the composition of the nanocapsules and notably by the presence of specific surfactants^28^. Being a medium chain triglyceride, Miglyol® has better nanoemulsifying property than long chain triglycerides^29,30^. In terms of surfactants, non-ionic surfactants with a hydrophilic/lipophilic balance (HLB) in the range of 12 to 18 were found to be preferable. Such compounds have a certain hydrophilicity and, hence, can be easily dispersed into the aqueous phase^31^. Tween®80, due to its low toxicity, is one of the widely used surfactants in self-emulsifying systems^32^. Solutol® was included in the formulation for two reasons: (1) presence of a PEG chain in its structure may provide stability to the nanocarrier^33^, and (2) it possesses the required high HLB (HLB = 14–16), along with an ability to inhibit p-glycoprotein pumps. The inhibition of this membrane pump in cancer cells would result in higher intracellular drug accumulation^29^.

The formation of HA NCs was achieved by attaching the HA shell to the CTAB-stabilized oily cores through electrostatic complexation. Nanocapsules coated with C12-HA were formed by direct association of the polymer with oily core without the use of a cationic surfactant. The dodecyl chains of C12-HA facilitated the deposition of the polymer onto the nanoemulsion interface, which might have been driven by hydrophobic interactions. The formation of the C12-HA coating is expected to result in an increased stability of the nanocapsule system^30^.

Stability in human plasma is a critical parameter in designing nano formulations for intravenous administration and targeted delivery. Often upon contact with the blood stream, nanocarriers suffer a disassembling or aggregation process, which may lead to their unfavorable biodistribution^34,35^. The HA nanocapsules described here were found to be stable in plasma and no particle aggregation was observed.

The anticancer agent DCX was selected as the drug candidate for our systems due to its high hydrophobicity and extensive history of clinical use^36,37^. We were able to encapsulate DCX efficiently within the oil core without changing the key physical parameters. The high encapsulation efficiency values (>85%) obtained for both types of HA formulations were not affected either by the type of polymer shell or the mechanism of nanocapsule formation. This suggests that the affinity of the drug for the oil phase was the main driving force behind the loading process. DCX-loaded nanocapsules formulated with HA and C12-HA showed a biphasic drug release profile; an initial burst release followed by a constant release (~70% release in 24 hrs.). This biphasic release profile has been typically observed in other HA-based nanocapsules, where the initial burst release has been attributed to the partition of the drug between the oil core and the aqueous external medium^38^. It should be noted that the initial burst was found to be reduced in the case of C12-HA nanocapsules compared to unmodified HA nanocapsules (from 55% to 45%). This behavior could be attributed to the entanglement of the hydrophobic chain at the interface of the nanocapsules, thereby retarding the diffusion of the drug from the oily core to the external medium. After this initial burst, the release process was similar for both formulations. With a pka_1_ = 2.82 and pka_2_ = 3.42, HA is negatively charged at pH above 4. Thus, it should maintain its ionized form in PBS medium (pH 7.4) and is expected to stabilize the nanocapsules in the physiological environment of blood. Furthermore, the hydrophobic chain in C12-HA may render the nanocapsule a more compact architecture, thus protecting DCX from being easily released.

A decrease in the cytotoxicity of C12-HA NCs was expected since the formulation of nanocapsules with the hydrophobically-functionalized HA eliminated the need for cationic surfactants. This was evident in the differences in the cellular toxicities of the two nanocapsules at the highest tested concentration (1 mg/mL). This finding correlates to the presence of the cationic surfactant CTAB in high concentration, which is in agreement with previous reports^39^. In addition, the marked difference in viability between the HA-based nanocapsules and the surfactant solution composed of Tween®80/Solutol/CTAB at 350 μg/mL suggests the beneficial effect of HA surrounding the surfactant layer as well as the correct isolation of the system from the free surfactants^40^. When loaded with DCX, both nanocapsules were taken up by A549 cells leading to inhibition of cancer cell growth. The high cellular uptake was observed for both HA and C12-HA nanocapsules loaded with the fluorescent dye, Nile red. As can be seen by confocal microscopy, strong fluorescent signals were detected in the cells cytoplasm for both nanocapsules prototypes in comparison to the free fluorophore. Furthermore, the fluorescent intensity was similar for both types of Nile red-loaded nanocapsules. This suggests that the functionalization of HA with the dodecylamide chain with a modest degree of substitution does not appear to affect HA’s binding affinity to CD44 receptors, or its ability to interact with the cell membrane. It might be attributed to the orientation of the lipophilic chain towards the oily phase and the hydrophilic anionic groups of HA chain towards the external aqueous phase^41^.

In summary, by carefully designing and synthesizing an amphiphilic HA derivative, we have successfully applied a solvent-free self-emulsification methodology to prepare a new class of nanocapsules consisting of an oily core and a shell made up of hydrophobically modified HA. The resulting nanocapsules were able to encapsulate hydrophobic drugs such as docetaxel quite efficiently and showed control over the release of the drug. These nanocapsules exhibited low cytotoxicity and demonstrated to be efficiently taken up by cancer cells to mediate a therapeutic effect.

## Methods

### Reagents

Sodium hyaluronate (Mw = 200 KDa) was provided by Sanofi Genzyme, USA. Caprylic/capric triglyceride (Miglyol®812) was a kind gift from Cremer, Germany. Polyoxyethylene sorbitan monooleate (Tween®80), hexadecyltrimethylammonium bromide (CTAB), Nile Red, DAPI and plasma (from human) were purchased from Sigma-Aldrich, Spain. Macrogol 15 hydroxystearate (former tradename Solutol®HS15, currently designated Kolliphor° HS15) was acquired from BASF, Germany. Dulbecco’s Modified Eagles Medium (DMEM) was purchased from Thermo Fisher Scientific, Spain. All other chemicals used were of reagent grade.

### Synthesis of dodecylamide-functionalized sodium hyaluronate (C12-HA)

An aqueous solution (concentration 10 mg/mL) of 200 mg of sodium hyaluronate was treated with 5 mL of Dowex 50WX8-400 resin (1.7 milliequivalents/mL, H^+^ form; freshly washed with water/methanol/water). After removing the resin by filtration, the resulting polymer solution was treated with 40% (w/w) aqueous tetrabutylammonium hydroxide solution until the pH of the solution became 12.0. The entire process of resin treatment and tetrabutylammonium hydroxide solution treatment was repeated twice. The final pH of the polymer solution was adjusted to 7.5–8.0 by bubbling with CO_2_ followed by bubbling with N_2_. The polymer solution was subsequently concentrated by tangential flow using a 30 KDa cut-off Pellicon XL Biomax filter cassette (EMD Millipore). The concentrate was lyophilized to dryness.

To the tetrabutylammonium hyaluronate (400 mg) were added 45 mL of DMF and 4 mL of monomethyl formamide. To the resulting polymer solution was added 8.8 mg of 2-bromo-1-ethyl pyridinium tetrafluoroborate dissolved in 1 mL DMF. After stirring for 1 h, a solution containing 12 mg of 1-aminododecane and 1.50 mL of triethylamine in 1 mL DMF was added to the reaction mixture. The mixture was stirred at room temperature for 48 hr. Subsequently the reaction mixture was added dropwise to 150 mL of acetone/tetrahydro-2-methylfuran (1:1) solution. The precipitate was collected, dissolved in 50 mL of deionized water, lyophilized to dryness and redissolved in 50 mL of deionized water.

The above solution was treated with 5 mL of Dowex 50WX8-400 resin and stirred for 10 min. The resin was filtered off and washed with deionized water. The filtrate was treated with 1 M NaOH until the pH was 12.0. The procedure was repeated two more times and the final pH was then adjusted to 7.5–8.0 by first bubbling CO_2_ followed by bubbling with N_2_. The solution was subsequently concentrated via tangential flow using a 30 KDa cut-off Pellicon XL Biomax filter cassette and the concentrate was lyophilized. The product (C12-HA) was characterized by ^1^H-NMR spectroscopy to confirm its structure and degree of substitution (See Supplementary Methods).

### Development of the self-emulsification method – primary emulsions

The self-emulsification method was initially optimized for the preparation of nanoemulsions, which was subsequently adapted to prepare HA-based nanocapsules.

Oil in water (o/w) nanoemulsions were prepared without organic solvents and heat, by using a single-step spontaneous emulsification process. Briefly, an oil phase (containing Miglyol®812 and Tween®80) was added to an aqueous phase (composed of water and Solutol®HS15). Miglyol®812 and Tween®80 were first mixed together and the mixture was subsequently poured into the aqueous phase and stirred at 900 rpm for 20 min. The optimization of the nanoemulsion composition was by varying the various components in the following manner:

- *Effect of Solutol*®*HS15 concentration in the aqueous phase*
  An oil phase composed of Miglyol®812 and Tween®80 (1:1 ratio w/w) was added under magnetic stirring to an aqueous phase (oil/aqueous phase ratio 1:2 v/v) containing different concentrations (2.5, 5, 15 and 25 mg/mL) of Solutol®HS15.
- *Influence of Miglyol*®*812*/*Tween*®*80 ratio*
  An oily phase composed of Miglyol®812 and Tween®80 at ratios (1:1, 1.5:1, 2:1, and 3.5:1 w/w) was prepared and poured into an aqueous Solutol®HS15 solution (oil/aqueous phase ratio 1:2 v/v). The concentration of Solutol®HS15 was kept either at 2.5 or 25 mg/mL.
- *Influence of oil*/*aqueous phase ratio*

The oily phase, composed of Miglyol®812/Tween®80 (1:1 ratio w/w) was added to the Solutol®HS15 solution at the concentration of 2.5 mg/mL, while the oil/aqueous phase ratios were varied between 1:2 and 1:30 (v/v).

### Preparation and optimization of HA nanocapsules

Using the optimized self-emulsification process described above, two types of HA-based nanocapsules were prepared by using aqueous solutions of sodium hyaluronate (HA) or dodecylamide-functionalized HA (C12-HA). These were called HA nanocapsules (HA NCs) and C12-HA nanocapsules (C12-HA NCs), respectively. To prepare HA NCs, the cationic surfactant CTAB was dissolved in the oil phase at different concentrations (0.05, 0.10 and 0.15 mg/mL). For both prototypes, the hyaluronan (HA or C12-HA) solutions were used three different concentrations (0.25, 0.5 and 1 mg/mL).

### Preparation of docetaxel-loaded HA nanocapsules

DCX was solubilized in Miglyol®812 and its solubility determined following the procedure of Saliou *et al*., with slight modifications^42^. DCX-loaded nanocapsules were prepared as described before by replacing Miglyol®812 with the DCX-Miglyol®812 solution. Loaded DCX was separated from the free drug by size exclusion chromatography (SEC). DCX was quantified by HPLC following the method of Rivera-Rodriguez *et al*.^43^. Yield and encapsulation efficiency (%) was calculated as described in Supplementary information (See Supplementary Methods).

### Preparation of fluorescent dye loaded HA nanocapsules

Nile red-loaded HA and C12-HA NCs were prepared as described before and the fluorescent probe was incorporated into the oil core. Encapsulated Nile red was separated from the free by SEC following the defined protocol.

### Characterization of nanocapsules

Characterization of HA-based NCs was carried out by measuring their mean particle size, polydispersity index (PDI) and zeta potential (ZP) using dynamic light scattering (DLS) (Zetasizer Nano-ZS, Malvern Instruments). Morphological analysis was carried out transmission electron microscopy (TEM).

### Assessment of stability of nanocapsules in human plasma

HA NCs and C12-HA NCs were diluted 1:10 (v/v) in human plasma for a period of 24 h, at 37 °C. At predetermined time intervals, samples were taken and particle sizes measured by the method described above.

### Measurement of physical stability of nanocapsules under storage conditions

For the assessment of the long-term stability of nanocapsules, samples were kept undiluted at 4 °C and stored for up to 6 months. Size and PDI were evaluated as described before.

### *In vitro* release assays

*In vitro* release (IVR) assays were assessed using a drug transfer method adapted from Bastiat *et al*.^25^. This method was optimized for the IVR profile of DCX from self-emulsifying HA-based nanocapsules under sink conditions. (See Supplementary Methods).

### ***In vitro*** cytotoxicity assays

The cell viability Alamar®Blue was utilized to assess the cytotoxicity of both empty and DCX-loaded HA-based NCs^44^. Serial dilutions of empty nanocapsules were added to A549 lung adenocarcinoma cells and incubated for 72 h. Similarly, cells were exposed to serial dilutions of free DCX, empty, and DCX-loaded HA-based NCs (DCX concentrations of 0.625, 1.25, 2.5, 5, 10 and 100 nM) for 48 h. After incubation, samples were withdrawn and replaced by fresh medium containing 5 mM AlamarBlue®. Fluorescence was measured at 530 and 590 nm (excitation and emission, respectively) using a microplate reader (Fluostar Omega, BMG Labtech, Germany). The relative cell viability (%) compared to control cells was calculated as the percentage of the fluorescence of the samples divided by the control (See Supplementary Methods).

### Cell uptake assay

Cellular uptake of Nile red-loaded HA NCs was studied in A549 cells. 60,000 cells/well were seeded in a cover glass and incubated with an appropriate volume of the formulation equivalent to 50 ng of fluorophore. After diluting in DMEM, the suspension media was left for 4 hr. At the end of this time, the cells were fixed, stained with DAPI, and were visualized by confocal microscopy (Leica, TCS SP5).

### Statistical analysis

Samples were prepared at least in triplicate and data presented as the mean ± standard deviation (SD). For *in vitro* cell assays, results are shown as the mean ± SD of the data obtained in two separate experiments with 3 replicates in each experiment (n = 6). Statistical evaluation of data was performed using the one-way analysis of variance (ANOVA). Tukey–Kramer multiple comparison test (GraphPad PRISM 5 software, La Jolla, CA, USA) was used to compare the significance of the difference between the groups, and a p < 0.05 was accepted as significant.

## Supplementary information

Supplementary Information

## Data Availability

All data generated and analyzed during this study are included in this published article and in the Supplementary Information section.
