# Supplementary material for: Self-assembled hyaluronan nanocapsules for the intracellular delivery of anticancer drugs
Source: Sci Rep. 2019 Aug 9;9:11565. doi: 10.1038/s41598-019-47995-8 (PMC6689112; doi:10.1038/s41598-019-47995-8)
Supplement: Supplementary file 1 — Supplementary Information [file 41598_2019_47995_MOESM1_ESM.pdf]

SUPPLEMENTARY INFORMATION

Self-assembled hyaluronan nanocapsules for the intracellular delivery of anticancer drugs

Ana Cadete [1,2], Ana Olivera [1,2], Magnus Besev [3], Pradeep K. Dhal [3], Lídia Gonçalves [4], António J. Almeida [4], Guillaume Bastiat [5], Jean-Pierre Benoit [5], María de la Fuente [6,7], Marcos Garcia-Fuentes [1,2], María José Alonso [1,2], Dolores Torres [2*]

[1] Nanobiofar Group, IDIS, CIMUS. University of Santiago de Compostela, Spain
[2]Department of Pharmaceutics and Pharmaceutical Technology, School of Pharmacy, University of Santiago de Compostela, Spain.
[3] Sanofi-Global R&D, Sanofi US, Waltham, USA
[4] Research Institute for Medicines (iMed.ULisboa), Faculty of Pharmacy, Universidade de Lisboa, Portugal
[5] Micro et Nanomedecines Translationnelles, MINT, Université Angers, INSERM 1066, CNRS 6021, 4 rue Larrey, Angers, France
[6] Nano-Oncology Unit, Translational Medical Oncology Group, Health Research Institute of Santiago de Compostela (IDIS), SERGAS, 15706 Santiago de Compostela, Spain.
[7] Cancer Network Research (CIBERONC), 28029 Madrid, Spain
* Corresponding author (email: dolores.torres@usc.es; +34609044859)

Supplementary Results

Supplementary Table S1. Influence of surfactant or polymer concentration on the size and zeta potential of the different formulations: anionic nanoemulsion, cationic nanoemulsions, HA NCs and C12-HA NCs.

Formulation	Surfactant/ coating material (mg/mL)	Conc. (mg/mL)	Size (nm)	ZP (mV)
**Anionic NE**	**-**	-	145 ± 1	-15 ± 2
**Cationic NEs**	**CTAB**	0.05	156 ± 2	-1 ± 1
		0.10	154 ± 2	+5 ± 1
		0.15	146 ± 3	+10 ± 1
**HA NCs**	**HA**	0.25	137 ± 11	-19 ± 1
		0.50	154 ± 2	-19 ± 2
		1.0	153 ± 6	-22 ± 4
**C12-HA NCs**	**C12-HA**	0.25	133 ± 11	-10 ± 1
		0.50	126 ± 5	-20 ± 2
		1.0	133 ± 3	-22 ± 3

The polydispersity index was 0.2 for all the formulations

Supplementary Table S2. Physical stability of self-emulsifying HA-based nanocapsules in storage conditions.

Time period	Size (nm)		PDI		ZP (mV)	
	**HA NCs**	**C12-HA NCs**	**HA NCs**	**C12-HA NCs**	**HA NCs**	**C12-HA NCs**
**First day**	134 ± 12	122 ± 3	0.2	0.2	-21 ± 1	-18 ± 1
**1 month**	138 ± 8	124 ± 6	0.2	0.2	-21 ± 1	-18 ± 1
**4 months**	136 ± 7	127 ± 6	0.2	0.2	-20 ± 1	-19 ± 1
**6 months**	137 ± 6	123 ± 1	0.2	0.2	-20 ± 1	-18 ± 1

Supplementary Methods

1. Synthesis of dodecylamide-functionalized sodium hyaluronate (C12-HA)

The degree of substitution of the dodecylamide group in the polymer chain was determined from the relative peak areas of the methyl groups of the acetamide group of HA and the methyl group of dodecylamide substituent. ^{1}H-NMR spectra were recorded at room temperature on a 400 MHz Varian NMR spectrometer using the residual proton resonance of the solvent as the internal standard. Chemical shifts are reported in parts per million (ppm).

2. Quantification of docetaxel loaded into HA-based NCs

The solubility of docetaxel (DCX) in Miglyol®812 was determined following the procedure of Saliou *et al.*, with slight modifications [25]. Briefly, an excess (2.5 mg) of DCX was poured in 0.5 mL of Miglyol®812 and stirred for 24 h at room temperature. The mixture was centrifuged 45 min at 20,000 g to separate the solution from the undissolved drug. The supernatant was collected, filtered and the concentration of DCX analyzed by HPLC (Elite LaChrom, VWR-Hitachi) using a reverse phase Zorbax® Eclipse XDB C8- 5 µm column (Agilent technologies).

DCX-loaded into nanocapsules was separated from the free drug by size exclusion chromatography (SEC) using CentriPure®P10 columns (EmpBiotech, Germany). Column preparation and equilibration were performed as described in the manufacturers' protocol. The elution profile of the free drug and the nanocapsules is shown in Figure 1. This method was initially validated for the free drug and empty nanocapsules (1mL of each, loaded separately) by the quantification of DCX alone and the physicochemical characterization of the nanocapsules in sequentially eluted volumes (400 µL). Being a small molecule (Mw = 808 Da), DCX gets entrapped within the column matrix and is eluted after the nanocapsules, without significant overlapping. After process optimization and validation, the following steps were followed to ensure the proper separation of loaded DCX from the free drugs: one mL of formulation was transferred to the column and eluted with water by simple gravity. The first 1.4 mL were discharged and the opalescent fraction, corresponding to 1.2 mL of the formulation, was collected and characterized as described along the paper. Nanocapsules yield was determined after lyophilizing and weighting the powder corresponding to 1 mL of the initial formulation and the collected elute. Encapsulation efficiency (EE%) was calculated by quantifying DCX concentration in the collected elute and in the initial formulation.

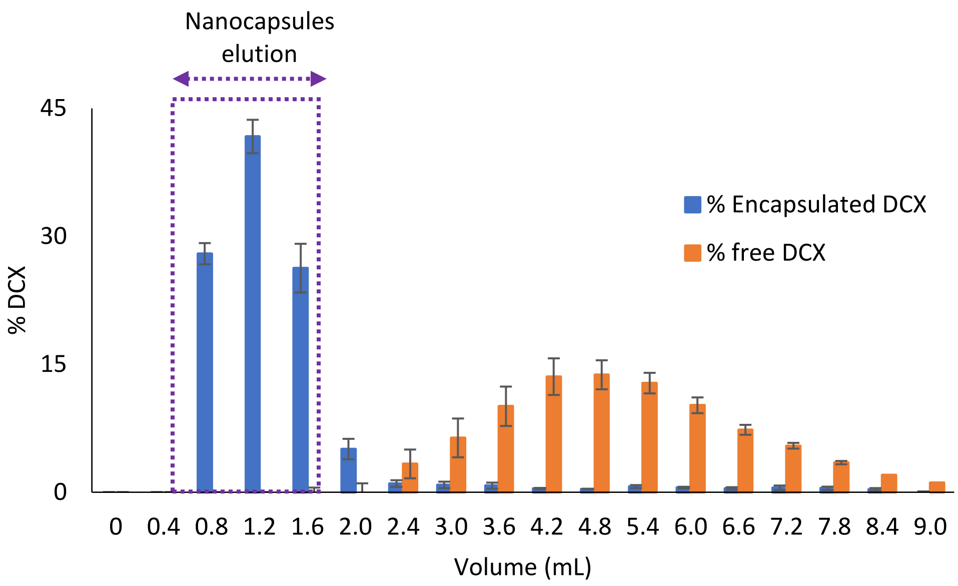

Supplementary Figure S1. Elution profile of free DCX and DCX-loaded HA-based nanocapsules by SEC.

3. In vitro release assays

Using 50 mL falcon tubes, DCX-loaded HA-NCs and C12-HA NCs were diluted under sink conditions in phosphate buffer saline (PBS) at 37ºC and placed inside a water bath incubator. At specific time points, 15 min, 3 h, 6 h and 24 h, 500 µL of sample were collected to an Eppendorf, mixed with an external oil compartment composed of Miglyol®812 (1:1 (v/v)), vortexed for 15 secs, and placed into a centrifuge for 30 min at 4000 rpm and 20ºC. After centrifugation, the oil and aqueous phases were separated. The nanocapsules suspension was characterized and the amount of drug in each phase was quantified by HPLC. The release pattern of drug was calculated with respect to the total amount of DCX in the release medium.

4. *In vitro* cytotoxicity assays

In vitro cytotoxicity of HA NCs and C12-HA NCs was evaluated by using the cell viability AlamarBlue® assay using A549 human lung adenocarcinoma cell line (ATCC® CCL-185™) for both empty and DCX-loaded HA-based NCs. The day before the experiment, A549 cells were cultured in sterile 96-well flat bottom plates in DMEM supplemented with 10% fetal bovine serum, 100 units of penicillin, 100 µg of streptomycin sulfate and 2 mM L-glutamine, at a cell density of $5x10^3$ cells/well. Cells were incubated at 37ºC and 5% CO_2. On the first day, medium was replaced by fresh medium containing the different formulations and each concentration was tested on six wells per plate. Cells were incubated for 72 h with empty nanocapsules and after that time, samples were withdrawn and replaced by fresh medium containing 5 mM AlamarBlue® and incubated for 3 h at 37ºC. Similarly, cells were exposed to serial dilutions of free DCX, blank, and DCX-loaded HA-based NCs (DCX concentrations of 0.625, 1.25, 2.5, 5, 10 and 100 nM) for 48 h and incubated with AlamarBlue®. Fluorescence was measured at 530 and 590 nm (excitation and emission, respectively) using a microplate reader (Fluostar Omega, BMG Labtech, Germany). The relative cell viability (%) compared to control cells was calculated as the percentage of the fluorescence of the samples divided by the control.
